# Rescue Therapy with a Proton Pump Inhibitor Plus Amoxicillin and Rifabutin for *Helicobacter pylori* Infection: A Systematic Review and Meta-Analysis

**DOI:** 10.1155/2015/415648

**Published:** 2015-05-25

**Authors:** Xiaoqun Liu, Hui Wang, Zhifa Lv, Youhua Wang, Ben Wang, Yong Xie, Xiaojiang Zhou, Nonghua Lv

**Affiliations:** Department of Gastroenterology, The First Affiliated Hospital of Nanchang University, Nanchang, Jiangxi 330006, China

## Abstract

*Background*. To conduct a systematic review and meta-analysis of clinical trials for eradication of *Helicobacter pylori* (*H. pylori*) that included a treatment arm with a proton pump inhibitor, rifabutin, and amoxicillin. *Materials and Methods*. We selected clinical trials that examined the efficacy of *H. pylori* eradication therapies and included a study arm using the test regimen from major medical literature databases and abstracts from major gastroenterology meetings. We also did subgroup and sensitivity analyses. *Results*. Twenty-one studies were included in systematic review. The total eradication rates of the test regimen were 70.4% by intent-to-treat (ITT) and 72.0% by per-protocol (PP) analyses. The pooled odds ratio (OR) was 0.55 using fixed effects model (*P* = 0.283) for the test regimen versus other triple regimens. The total eradication rates were 68.4% for the test regimen and 81.9% in the control group by ITT, while the OR was 1.08 using random effects model (*P* = 0.019). The pooled eradication rate was 66.4% for the test regimen and 67.4% for the control group by ITT. The total adverse effects incidence were 25.1% for the test regimen. *Conclusions*. The test regimen for *H. pylori* rescue therapy may be not superior to control regimens in efficacy.

## 1. Introduction

*Helicobacter pylori* (*H. pylori*) infection affects approximately 50% of the adult population worldwide [1] and contributes to several upper gastrointestinal tract diseases, such as chronic gastritis, peptic ulcers, and gastric cancer [2, 3]. Standard triple therapy, a PPI plus two of these three antibiotics, amoxicillin, metronidazole, and clarithromycin, has been widely used for* H. pylori* eradication for many years due to good compliance and high efficacy [4, 5]. However, the prevalence of antibiotic-resistant strains of* H. pylori* is increasing, in particular strains resistant to clarithromycin and metronidazole [6]. A recent study showed that the resistance rates of* H. pylori* to clarithromycin and metronidazole were more than 80% and 20%, respectively [7]. This resistance results in eradication failure in 5–30% of patients positive for* H. pylori*. Eradication of* H. pylori* fails in some patients even after two or three different therapies. These patients constitute a therapeutic challenge. The Maastricht IV Consensus Report recommended that treatment for these patients should be guided by antimicrobial susceptibility testing, whenever possible [8]. Sometimes antimicrobial susceptibility testing is not locally available. Therefore, the results of bacterial sensitivity testing cannot be obtained and cannot be used to guide therapy in all patients.

Recent studies have shown that the prevalence of* H. pylori* resistant to rifabutin and amoxicillin was low, so a regimen with the combination of rifabutin and amoxicillin might achieve high eradication rates for* H. pylori* infection. This regimen was recommended for rescue therapy in some consensus reports [5, 7, 8]. However, clinical outcomes for this regimen have been mixed. A study by Borody et al. showed that a regimen with pantoprazole, amoxicillin, and rifabutin achieved a 90.7% eradication rate [9]. However, Gisbert et al. recently reported that the eradication rate was only 50% by intention-to-treat analysis (ITT) when the combination of PPIs, rifabutin, and amoxicillin was used as fourth-line therapy [10].

A systemic review by Gisbert et al. showed that rifabutin-containing rescue therapy constitutes an encouraging strategy after multiple (usually three) previous eradication failures [11]. But recent study showed that rifabutin-containing rescue therapy cloud not achieve satisfactory eradication rates for* H. pylori* infection [10]. So, we conducted a systematic review and meta-analysis in order to evaluate the efficacy of a triple regimen consisting of PPI, rifabutin, and amoxicillin in patients for whom one or more consecutive eradication regimens had failed. We also compared the efficacy of this regimen with that of other regimens.

## 2. Methods

### 2.1. Search Strategy and Study Selection

Using the Preferred Reporting Items for Systematic Reviews and Meta-Analyses (PRISMA) statement guideline, we developed this meta-analysis [12, 13]. The following databases: PubMed (through June 2014), Embase (1946 to June 2014), the Cochrane Central Register of Controlled Trials (Issue 6, 2014), and the Science Citation Index (SCI) (1945 to June 2014), were searched using medical subject headings and text terms: (*Helicobacter pylori* OR* H. pylori*) AND amoxicillin AND rifabutin. The abstracts of major gastroenterological society meetings, such as the Digestive Disease Week from the American Gastroenterological Association (DDW) (2008–2014) and European Helicobacter Study Group (2008–2013), were also searched. In addition, authors of some of the identified studies were asked to provide unpublished clinic trial results. In addition, we searched the clinicaltrials.gov website for registered randomized clinical trials for unpublished data. References for the identified trials were reviewed for relevant studies.

### 2.2. Inclusion Criteria

The inclusion criteria were the following. (1) The study must be a clinical trial; (2) the subjects must have endoscopic findings and symptoms at the time of enrollment; (3) there must be confirmation of* H. pylori* eradication by urea breath test or histology or* H. pylori* stool antigen at least four weeks after therapy; and (4) the eradication regimens must include PPI, rifabutin, and amoxicillin regimen in a treatment arm.

### 2.3. Exclusion Criteria

Studies were excluded under the following circumstances. (1) The eradication date could not be determined; (2) the article or abstract was written in a language other than English or Chinese, unless a translation to one of these two languages was available; (3) rifabutin and amoxicillin were not included in the same experimental group.

### 2.4. Data Extraction and Quality Assessment

Standardized data abstraction sheets were prepared. Two authors (X. Liu and H. Wang) independently extracted the relevant data and entered it into standardized data abstraction sheets. The recorded data included the following: (1) the study characteristics and type, (2)* H. pylori* treatment regimens, (3) duration of treatment, (4) location of the trial, (5) date of publication, (6) the number of enrolled patients, (7) the average age of the enrolled patients, (8) diagnostic methods for detecting* H. pylori* infection before enrolling and after completing study, (9) eradication rates by intention-to-treat analysis (ITT) and preprotocol (PP), (10) rates of successful and failed eradication, and (11) total adverse effects.

Two reviewers (X. Liu and H. Wang) independently assessed the quality of the studies that met the inclusion criteria. Discrepancies were resolved by consulting a third reviewer (Y. Xie). The reviewers were not blinded to the authors or journal. To avoid duplication of data, if a trial was published in more than one place by the same authors or institutions, the most recent or most informative study was included. The quality of randomized controlled trials studies was assessed by the Cochrane collaboration's tool for assessing risk of bias. And we used Methodological Index for Nonrandomized Studies (MINORS) to assess the nonrandomized studies [34].

### 2.5. Statistical Analysis

The primary outcome for the systematic analysis was the efficacy of the regimen with PPI, rifabutin, and amoxicillin. The secondary outcome was the safety of the combination of PPI, rifabutin, and amoxicillin. We measured* H. pylori* eradication efficacy by calculating the odds ratio (OR) with 95% confidence intervals (CIs). When data could be combined, the meta-analyses were performed. If the data could not be combined, the results were described based only on ITT and adverse events. Heterogeneity between studies was assessed by the *Q*-test and the *I* ^2^ statistic. A higher *I* ^2^ statistic indicates greater heterogeneity between studies. We pooled the ORs for all studies into a summary OR, using fixed effects model and random effects model, based on inverse variance methods. The fixed effects model was used when the heterogeneity between studies did not have statistical significance; otherwise, the random effects model was employed. If the data were shown to be heterogeneous, we searched for sources of heterogeneity by subgroup analysis. A *Z*-test also was employed for estimating the pooled effects. We employed funnel plot, Egger's test, and Begg's test to assess the publication bias where a two-sided *P* value of 0.10 or less was significant. We used Comprehensive Meta-Analysis Software (Version 2) to perform the statistical analysis.

## 3. Results

### 3.1. Description of the Studies

**Figure 1:**
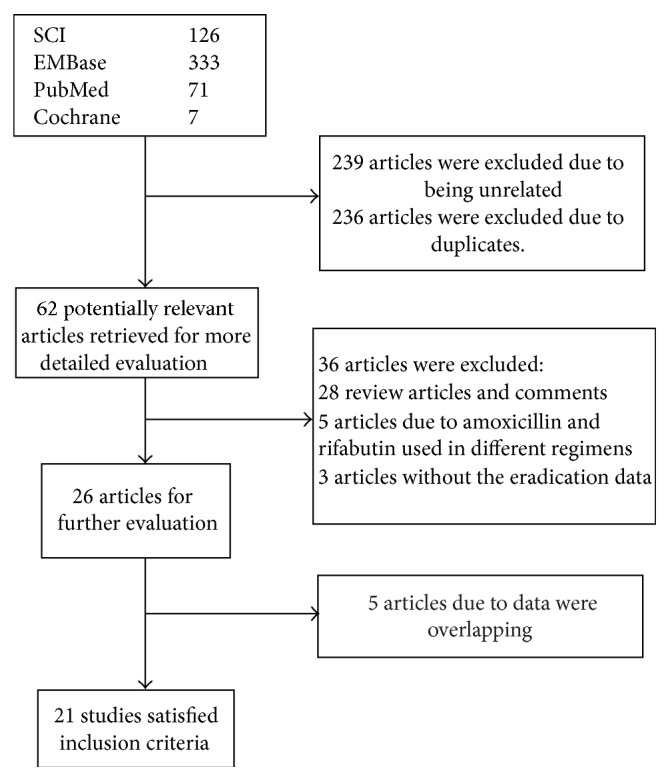
Flow diagram of trials identified and selected.

The bibliographical search yielded 537 articles from PubMed, the Cochrane Central Register of Controlled Trials, Embase, and SCI. We excluded 239 articles that were unrelated to our study and 236 due to duplication. We selected 62 articles for detailed evaluation. Among the selected studies, 28 review and comment articles were excluded. Five articles were excluded because amoxicillin and rifabutin were not used in the same treatment arm [35–39]. Three articles were excluded because the eradication date could not be determined [40–42]. Twenty-six articles met the inclusion criteria, and five articles were excluded because the data overlapped with other included studies [10, 43–46]. Ultimately, 21 studies were included in the systematic review and meta-analysis [9, 14–33] (Figure 1).

### 3.2. Eradication Rates

The total eradication rates for* H. pylori* were calculated for the 21 selected studies. The total eradication rates of the regimen with PPI, amoxicillin, and rifabutin were 70.4% (771/1095) by ITT and 72.0% (771/1071) by PP.

### 3.3. Meta-Analysis

#### 3.3.1. Triple Regimens Containing Amoxicillin and Rifabutin versus Other Triple Regimens

**Figure 2:**
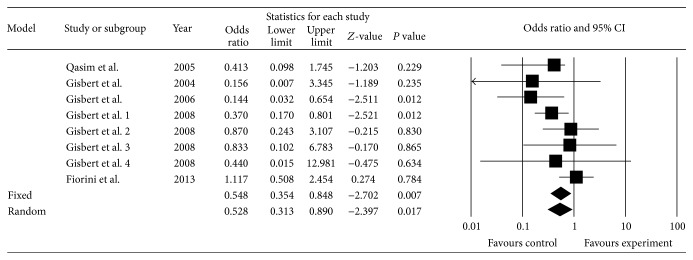
Triple regimens with PPI, amoxicillin, and rifabutin compared with other triple regimens.

Five studies [14, 17, 18, 20, 21] (Table 1), which included eight subgroups, were included in the pooled analysis of triple regimens containing PPI, amoxicillin, and rifabutin (experimental group) versus other triple regimens (control group). The total* H. pylori* eradication rates were 68.4% (158/231) in the experimental group and 81.9% (222/271) in the control group by ITT analysis, respectively. The pooled OR was 0.55 (95% CI: 0.35, 0.85) using fixed effects model (*I* ^2^ = 18.59%, *P* = 0.283; Figure 2). The result showed that the eradication rate of combination of PPI, rifabutin, and amoxicillin was inferior to other triple regimens. When we omitted the study with the greatest weight from the analysis [21], the OR was 0.42 (95% CI: 0.14, 1.27) using random effects model (*I* ^2^ = 82.73%, *P* < 0.001). When the study conducted by Fiorini et al. was omitted [14], the OR was 0.40 (95% CI: 0.24, 0.67).

We performed subgroup analyses using third- or fourth-line regimens in the control group. The third-line subgroup achieved a lower eradication rate than the control group (OR = 0.38, 95% CI: 0.22–0.66, *P* = 0.001). The eradication rate of the combination of PPI, rifabutin, and amoxicillin was not superior to the combination of PPI, levofloxacin, and amoxicillin. The difference between these groups was not significant (OR = 0.50, 95% CI: 0.22–1.20, *P* = 0.118).

#### 3.3.2. Triple Regimens Containing Amoxicillin and Rifabutin versus Quadruple Regimens Containing Bismuth

**Figure 3:**
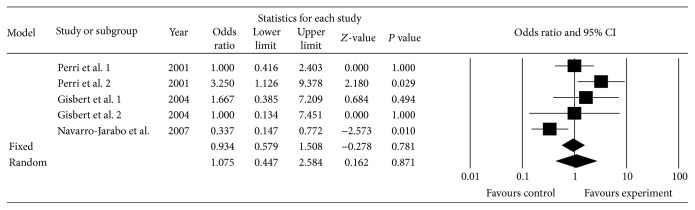
Triple regimens with PPI amoxicillin rifabutin compared with quadruple regimens.

Three studies, including five subgroups [15, 19, 20] (Table 2), were selected to compare the regimen with PPI, amoxicillin, and rifabutin (experimental group) with the regimen using PPI, bismuth, tetracycline, and metronidazole (control group). The pooled eradication rate was 66.4% (99/149) by ITT in the experimental group and 67.4% (85/126) by ITT in control group. The pooled OR was 1.08 (95% CI: 0.45, 2.58) by random effects model (*I* ^2^ = 66.0%, *P* = 0.019). There was no statistical significance between the groups (*Z* = 0.16, *P* = 0.87) (Figure 3). When we omitted the study with the greatest weight [15], the pooled OR was 1.13 (95% CI: 0.33, 3.87) by random model (*I* ^2^ = 74.4%, *P* = 0.008).

Due to the evidence of heterogeneity, we performed subgroup analyses depending on duration of treatment (7, 10, or 14 days), second- or third-line treatment, and control group regimen (quadruple treatment). The result showed that the eradication rate of the 7-day treatment was lower than quadruple therapy containing bismuth (OR = 0.34, 95% CI: 0.15–0.77, *P* = 0.010). When the rifabutin was used as a part of second-line therapy, the eradication efficacy was inferior to quadruple therapy with bismuth (OR = 0.34, 95% CI: 0.15–0.77, *P* = 0.010).

### 3.4. Data Not Included in Meta-Analysis (Table 2)

The regimen with amoxicillin, rifabutin, and PPI was used as third-line therapy in four studies [23, 24, 28, 33]; the pooled eradication rate was 64.2% (79/123). The study by Gisbert et al. [23] showed that the regimen with standard dose PPI, amoxicillin 1000 mg, and rifabutin 300 mg achieved 79% eradication rate by ITT in 14 patients with* H. pylori* infection after two eradication failures. Five side effects were observed including abdominal pain, nausea, vomiting, and* Candida* infection. The study by González Carro et al. [24] found that the eradication rate for regimens with a PPI plus amoxicillin and rifabutin 150 mg was 60% for* H. pylori* infection. The incidence of adverse effects was 4%. Zullo et al. [28] showed that triple therapy containing amoxicillin and rifabutin achieved 76.5% eradication rate. However, the study only included 17 patients. Only two adverse effects were observed. Moon et al. [33] reported that triple therapy containing amoxicillin and rifabutin attained an acceptable eradication rate (84.3% by ITT, 43/51), and addition of a high dose PPI achieved higher efficacy (94.7% versus 78.0% by ITT).

There was only one study that used rifabutin as fourth-line therapy [31]. The eradication rate was 52% (80/153). These results suggested that the regimen with a PPI plus amoxicillin and rifabutin did not achieve a satisfactory eradication rate. Adverse effects were reported in 51 (33%) patients. Leukopenia and thrombocytopenia were seen in eight patients and resolved spontaneously in all cases after treatment ended.

In seven studies, the regimen with amoxicillin, rifabutin, and PPI was used as rescue therapy [9, 25–27, 29, 30, 32]. The pooled eradication rate was 81.7% (294/360) demonstrating the strong efficacy of regimens with amoxicillin and rifabutin. The two studies conducted by Perri et al. [26, 30] showed that the eradication rate of the regimen with PPI, amoxicillin, and rifabutin was 78.6% and 70.7% by ITT, respectively. Two studies conducted by Borody et al. [9, 32] suggested that a regimen with PPI, amoxicillin, and rifabutin could achieve satisfactory eradication of* H. pylori*. The study by Van der Poorten and Katelaris [29] showed that the triple treatment with PPI, rifabutin, and amoxicillin achieved a 71.5% eradication rate by ITT. Another study showed that the regimens with amoxicillin and rifabutin attained only a 62.5% eradication rate by ITT [25]. Bock et al. reported that the regimen with PPI, amoxicillin and rifabutin achieved 72% eradication rate by ITT [27]. Miehlke et al. reported the triple regimen containing PPI, amoxicillin, and rifabutin achieved a 74% (54/73) eradication rate by ITT [16].

### 3.5. Adverse Effects and Compliance

Within the 21 studies selected, 17 studies examined adverse effects of treatment. These adverse effects included [9, 14–16, 18–20, 22–24, 26, 28–31] abdominal pain, vomiting, dysgeusia, odynophagia, and diarrhea. Leukopenia, a serious adverse event, was reported for patients in three studies [18, 22, 31]. Among these three studies, in two studies patients were treated with rifabutin, amoxicillin, and PPI [22, 31], while the other adverse event was in a study where patients were treated with levofloxacin (500 mg daily), amoxicillin (1 g daily), and omeprazole (20 mg daily) for ten days [18]. The rate of total adverse effects was 25.1% (117/467) in regimens with a PPI plus amoxicillin and rifabutin.

**Figure 4:**
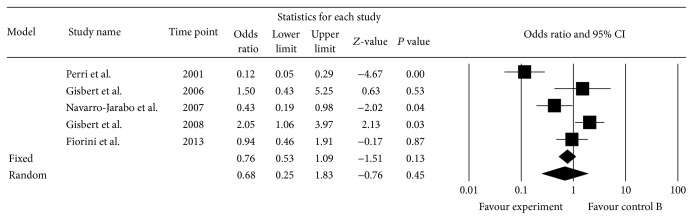
Total adverse effects.

Five studies included in the meta-analysis provided information concerning adverse effects [14, 15, 18, 19, 24]. The incidence of total adverse effects was 21.7% (77/318) in the experimental group and 24.2% (140/474) in the control group. The pooled OR was 0.68 (95% CI: 0.25–1.83) by a random effect model (*I* ^2^ = 85.99%, *P* = 0.000) (Figure 4).

Overall, patients in six studies were fully adherent to their antibiotic therapy protocol [9, 14, 17, 21, 23, 30]. Other studies had varying degree of follow-up bias. In seven clinical studies, three showed a trend toward higher compliance rates with rifabutin-containing regimens than with conventional therapeutic regimens [15, 18, 19]. The other four studies showed no significant difference in compliance between the two regimens or compliance was not reported [14, 16, 17, 31]. For patients who dropped out of the trials, the most common reason was adverse events related to the treatment. Other reasons included inability to return for follow-up visits and inability to consume the medication provided. In one of the articles selected, a difference in compliance to therapy after primary resistance and secondary resistance was reported [22]. Compliance after secondary resistance was better than that after primary resistance.

### 3.6. Risk of Publication Bias

**Figure 5:**
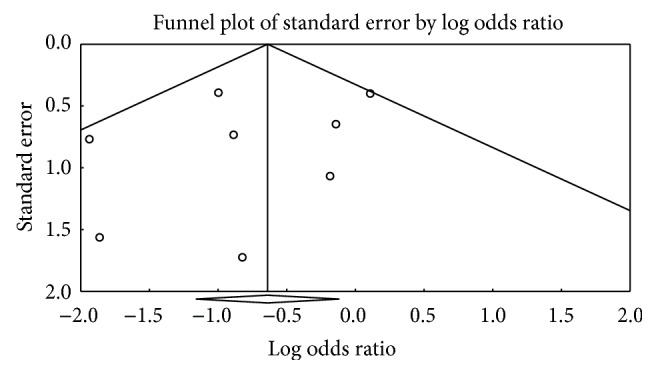
The funnel plot of triple regimen containing amoxicillin and rifabutin compared with other triple regimens.

**Figure 6:**
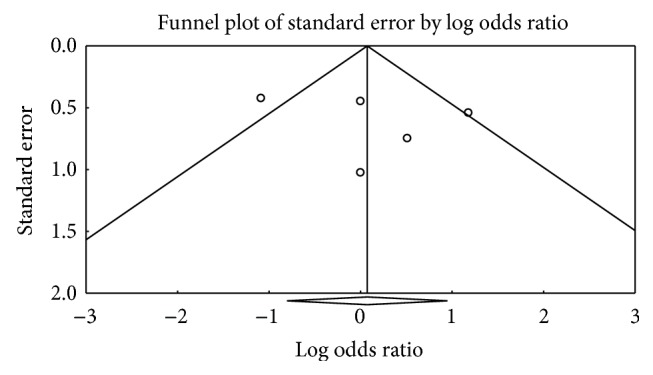
The funnel plot of triple regimens containing amoxicillin and rifabutin compared with quadruple regimens.

Funnel plots of both meta-analyses appeared nonsymmetrical. However, Egger's test and Begg's test did not indicate statistically significant differences (Figures 5 and 6).

## 4. Discussion

The antibiotic resistance of* H. pylori* is different in some regions of the world, and therefore the current recommended first-line and second-line therapies are different in guidelines for different regions [8, 47]. In addition, there is no standard regimen for* H*.* pylori* rescue therapy [8, 47]. The following regimens: (1) regimens including rifabutin or quinolone, (2) quadruple therapy containing bismuth, (3) dual therapy, or (4) tailored therapy, might be considered as alternatives for rescue therapy [8, 48, 49].

Rifabutin, an antimycobacterial agent, is a promising agent due to its high* in vitro* bactericidal activity against clinical isolates of* H. pylori* [50]. Some studies have shown that the resistance to rifabutin in* H. pylori* is rare [51–53]. In addition, rifabutin is chemically stable in the gastric environment and its antibacterial activity is unlikely to be affected by the gastric acid [54–56]. Rifabutin achieves a high intracellular concentration leading to marked activity. Thus, the combination of rifabutin and amoxicillin should show additive antimicrobial effects, high* in vivo* effectiveness, and good tolerability [14, 16]. However, some studies have shown that the combination of rifabutin and amoxicillin did not achieve higher eradication efficacy than other rescue therapies [17]. There is also some controversy about whether or not rifabutin can be safely used for* H. pylori* due to the possibility of cross-resistance to rifampicin and induction of myelosuppression [57–59].

Our results suggested that the eradication effectiveness of rescue therapy with regimens including the combination of rifabutin and amoxicillin was lower than that of other triple therapies (68.4% versus 81.9% eradication rate). In addition, the effectiveness of the combination was not superior to the combination of amoxicillin and levofloxacin according to sub-nalysis. The meta-analysis in the current study also indicated that the effectiveness of the combination of amoxicillin and rifabutin was similar to the quadruple therapy that included a PPI and amoxicillin. Our results also confirmed that the efficacy of the combination of rifabutin and amoxicillin was inferior to quadruple therapy containing bismuth, according to a 7-day subgroup and second-line subgroup analyses. We concluded that a regimen with PPI, rifabutin, and amoxicillin for* H. pylori* infection might not be the optimal selection for rescue therapy after one or more eradication failures. Our finding might be inconsistent with the previous systemic review [11]; the main reason might be that the studies included in our meta-analysis only contained triple regimens with PPIs, rifabutin, and amoxicillin. However, rifabutin-containing rescue therapy might be a viable strategy after multiple previous eradication failures with key antibiotics such as amoxicillin, clarithromycin, metronidazole, tetracycline, and levofloxacin [31, 49, 51–53].

The frequency of adverse effects in the rifabutin treatment group in* H. pylori* studies was 25.1% in the current study. Uveitis has recently been reported in patients treated with a combination of rifabutin and other antimycobacterial drugs [60–63], but this complication was not reported during* H. pylori* therapy in the current study. Myelotoxicity is the most significant adverse effect of rifabutin during* H. pylori* therapy; although this complication is rare, the current study showed that myelotoxicity was reported in only four of the selected studies. To date, all patients reported to have leukopenia have recovered uneventfully in a few days. There have been no reports of infection or other adverse outcomes related to leukopenia during* H. pylori* treatment [64–66].

Our results showed that the adverse effects of regimens containing amoxicillin and rifabutin were 21.7% and were lower than control group (24.2%) in meta-analysis. The adverse effects were similar between experiment and control group, and the difference was not statistically significant. This indicates that the regimen containing with rifabutin and amoxicillin is safe.

However, the widespread use of rifabutin for* H. pylori* eradication therapy also raises some concerns since rifabutin is an established antimycobacterial drug. Multidrug resistant strains of* Mycobacterium tuberculosis* are rapidly increasing, and rifabutin should be used very carefully for* H. pylori* to avoid further acceleration of development of resistance. At present, the rifabutin should be restricted to patients who have experienced one or more* H. pylori* eradication therapy preferably due to resistance of* H. pylori* to key antibiotics [49, 53, 62, 66].

## Figures and Tables

**Table 1:** Characteristics of studies included meta-analysis.

Study, year	Age	Location	Control group (day)^†^	Rifabutin group (day)^†^	*Hp* infection diagnosis/rechecking^●^	Eradication rate by ITT (control/rifabutin)^Ф^	Eradication rate by PP (control/rifabutin)^◊^	Side effects (control/rifabutin)	Therapy^#^	Risk of bias assessment^∗^	MINORS score
Fiorini et al. [14], 2013	Adults	Italy	EAL-10	ERA-12	H/13C-UBT	90.0% (118/131)/88.6% (93/105)	92.2% (118/128)/90.3% (93/103)	15.3% (20)/0	Rescue	—	19

Navarro-Jarabo et al. [15], 2007	Adults	Spain	OBTM-7	ORA-7	H/UBT	70.40% (38/54)/44.40% (20/45)	77.10% (38/48)/46.50% (20/43)	64% (35)/44% (20)	Second	YYYNYY	—

Miehlke et al. [16], 2006	Adults	Germany	OA-14	ERA20	C/(H/C13-UBT)	69.50% (50/72)/74% (54/73)	74.60% (50/67)/78.30% (54/69)	5%/2%	Second or third	YYUNYY	—

Qasim et al. [17], 2005	Adults	Ireland	PPI-FA-7	PPI-RA-10	C13-UBT/C13-UBT	60% (6/10)/38% (13/34)	—/—	0/0	Third	—	15

Gisbert et al. [18], 2006	Adults	Spain	OAL-10	ORA-10	C13-UBT/C13-UBT/	85% (17/20)/45% (13/16)	81.00% (9/20)/45% (13/16)	10/12	Third	—	18

Perri et al. [19], 2001	Adults	Italy	PBMT-10	PRA150-10PRA300-10	C13-UBT/C13-UBT	67% (30/45)/86.6% (39/45)	73% (30/41)/86.6% (39/45)	21/5	Rescue	YYYNYY	—

Gisbert et al. [20], 2004	Adult	Spain	OAC-7, OBMT-7, OBAC-14	ORA-14	C13-UBT/C13-UBT	70.5% (24/34)/71.4% (10/14)	70.5% (24/34)/72.30% (10/13)	10	Third	—	14

Gisbert et al. [21], 2008	Adults	Spain	RBC-MT-7, OLA-(7–10),	PRA-10	C13-UBT/C13-UBT	Third: 58% (7/12), 76.7% (56/73)/54.9% (28/51)Fourth: 100% (2/2), 75% (9/12)/71.4% (5/7)	—	—	Third or fourth	—	12

^†^E: esomeprazole, O: omeprazole, PPI: proton pump inhibitor, P: pantoprazole, RBC: ranitidine bismuth citrate, B: bismuth, A: amoxicillin, L: levofloxacin, R: rifabutin, T: tetracycline, M: metronidazole, F: furazolidone, and C: clarithromycin.

^●^C: culture; UBT: urea breath test; H: histology.

^Ф^ITT: intention to treat; ^◊^PP: per protocol.

^∗^The six letters in the risk of bias assessment columns stand for bias of sequence generation, allocation concealment, blinding of participants, blinding of outcome assessors, incomplete outcome data, and selective outcome reporting, respectively. Y: a low risk of bias; N: a high risk of bias; U: an uncertain risk of bias.

^#^Rescue: rescue therapy, second: second-line therapy, third: three-line therapy, fourth: fourth-line therapy.

“—”: not reported.

**Table 2:** Characteristics of studies without included meta-analysis.

Study, year	Age	Location	Regimen^†^	Duration (day)	*H*. *pylori* infection diagnosis/rechecking^●^	Eradication rate by ITT^Ф^	Eradication rate by PP^◊^	Side effects	Discounting	Therapy^∗^	MINORS score
Toracchio et al. [22], 2005	Adult	Italy	PRA	10	RUT and C13-UBT	Group 1: 87.5% (366/420) Group 2: 78.5% (82/104)	Group 1: 100% (366/366) Group 2: 82.2% (82/10)	1	—	First or second	18

Gesbert et al. [23], 2003	Adult	Spain	ORA	14	C13-UBT/C13-UBT	79% (11/14)	79% (11/14)	5	0	Third	13

Borody et al. [9], 2006	Adult	Australia	LRA	12	C13-UBT	90.8% (118/130)	90.8% (118/130)	52	—	Second, third, fourth	14

González Carro et al. [24], 2007	Adult	Spain	PRA	10	C13-UBT/C13-UBT	61% (55/92)	62.20% (55/90)	2	2	Third	13

Veldhuyzen van Zanten et al. [25], 2010	Adult	Canada	PPI-RA	7	H, C/H, UBT	63% (10/16)	67% (10/15)	—	1	Rescue	9

Perri et al. [26], 2000	Adult	Italy	PRA	7	C13-UBT/C13-UBT	71.00% (29/41)	74.00% (29/39)	1	2	Rescue	13

Bock et al. [27], 2000	Adult	Germany	LRA	7	C13-UBT/C13-UBT	72% (18/25)	86% (18/21)	0	4	Rescue	10

Zullo et al. [28], 2010	Adult	Italy	ORA	10	C13-UBT/C13-UBT	76.40% (13/17)	79.50% (13/16)	10	3	Third	14

Van der Poorten and Katelaris [29], 2007	Adult	Australia	PPI-RA	10	H, RUT, C13-UBT/C13-UBT	72% (48/67)	76% (48/63)	7	4	Rescue	10

Perri et al. [30], 1998	Adult	Italy	RAP	7	C13-UBT/C13-UBT	79.00% (22/28)	79.00% (22/28)	1	0	Rescue	14

Gisbert et al. [31], 2014	Adult	Spain	PPI-RA	10	—/C13-UBT	52%	53%	51	10	Fourth	NA

Borody et al. [32], 2012	Adult	Australia	ORA	14	—/UBT	92% (52/56)	94% (52/55)	—	4	Rescue	NA

Moon et al. [33], 2012	Adult	Korea	LRA	12	—	92.2% (47/51)	94% (47/50)	—	1	Third	15

^†^E: esomeprazole, O: omeprazole, PPI: proton pump inhibitor, P: pantoprazole, L: lansoprazole, A: amoxicillin, and R: rifabutin.

^●^C: culture; UBT: urea breath test; H: histology; RUT: rapid urease test.

^Ф^ITT: intention to treat; ^◊^PP: per protocol.

^∗^Rescue: rescue therapy, second: second-line therapy, third: three-line therapy, fourth: fourth-line therapy.

“—”: not reported; NA: not assessed.

Sign:

Toracchio et al. (2005) [22]: patients in group 1 without previous exposure to triple therapy; patients in group 2 had already received one course of triple therapy.

Two abstracts, Gisbert et al. (2014) [31] and Borody et al. (2012) [32], quality cannot be assessed because we do not know the detailed method of studies.

## Strengths and Limitations

Study selection, data extraction, and evaluation were performed by two reviewers. Most of the clinical trials used the current systemic review and meta-analysis was published in English. We comprehensively analyzed the efficacy of the regimens with PPIs, rifabutin, and amoxicillin for* H. pylori* infection and performed sensitivity analyses and subgroup analyses to make the outcomes of our meta-analysis more reliable.

Several faults might limit the validity and generalizability of this meta-analysis. First, most of the studies included in our meta-analysis were retrospective studies and that might affect our results although we performed sensitivity analysis to assess the reliability of the results. Second, there was some obvious heterogeneity in the meta-analysis, although we conducted subgroup analysis to decrease the effects of this heterogeneity. Thirdly, the studies included in our meta-analysis mostly were nonrandomized clinical trials, and the quality of some studies might be low due to small sample size and the lack of blinding. Finally, we asked some authors for unpublished data, so these data could lead to the introduction of bias.

In summary, a regimen with PPI, amoxicillin, and rifabutin for* H. pylori* infection does not appear to be superior to other regimens whether used as a primary or rescue treatment. The regimen might be a useful alternative when* H. pylori* is resistant to some key antibiotic agents, such as clarithromycin, metronidazole, tetracycline, and levofloxacin.
